# Thermal Desorption of 2,4,6-Trichloroanisole from Cork

**DOI:** 10.3390/foods12183450

**Published:** 2023-09-15

**Authors:** Susana Monteiro, Nenad Bundaleski, Paulo Lopes, Miguel Cabral, Orlando M.N.D. Teodoro

**Affiliations:** 1 CEFITEC–Center of Physics and Technological Research, Department of Physics, Nova School of Sciences and Technology, 2829-516 Caparica, Portugal; 2 Amorim Cork, S.A., Rua dos Corticeiros, 850, 4536-904 Santa Maria de Lamas, Portugal

**Keywords:** cork stoppers, TCA, vacuum, mass spectrometry, calibration method, TCA extraction

## Abstract

While extensive efforts have been made over the past two decades to understand how cork becomes contaminated by 2,4,6-trichloroanisole (TCA), the nature of its bond to cork remains unclear. A deeper understanding of this interaction is crucial in designing processes to effectively remove TCA from cork stoppers. This study presents an investigation into the thermal desorption of TCA from cork under vacuum conditions. To facilitate detection by a quadrupole mass spectrometer, samples were artificially contaminated with sufficient TCA. A calibration system was developed to determine the absolute rate of TCA released from the cork. Desorption spectra revealed two peaks at 80 °C and 170 °C. Despite the known variability of cork, repeated measurements demonstrated reasonable repeatability. The low-temperature peak decreased with time and after preheating the sample to 50 °C. It is proposed that the high-temperature peak corresponds to TCA bonded to the cork material. Experiments with naturally contaminated cork stoppers revealed a significant reduction in the amount of releasable TCA following a vacuum-heating process. This study provides an insightful discussion on the adsorption of TCA on cork and proposes an estimate for the adsorption energy. Furthermore, it discloses a process capable of removing TCA from natural cork stoppers.

## 1. Introduction

Cork has many advantages for wine closures. But, it also introduces some concerns. Wine contamination with fungal aromas is one of the problems faced by wine producers and cork industries. 2,4,6-trichloroanisole (TCA) was identified as the main compound responsible for causing sensory faults in wine; it originates from cork and reduces the quality of the wine [1,2]. The origin of the contamination of cork stoppers occurs, for the most part, in the forest, when halophenols are converted into haloanisoles by fungal action [3]. TCA can also be transferred from one contaminated stopper to another during the manufacturing process (cross contamination), as demonstrated in previous works [4,5].

Several technologies have been developed to reduce/eliminate TCA from cork stoppers. Recently, Tarasov et al. reviewed many of the processes developed to clean contaminated cork. Some of the techniques have been shown to have limited effectiveness in decreasing TCA or are not suitable for use on an industrial scale [6,7].

To design processes to extract TCA from cork, it is essential to understand how strongly TCA molecules are bonded to the cork surface. Insights into this matter can be obtained by an Evolved Gas Analysis coupled with mass spectrometry. Samples are heated at a constant rate while the released gases are monitored by a mass spectrometer [8]. If samples are heated under a vacuum, then desorbed gases can be directly analyzed by a residual gas analyzer. In surface science, this technique is commonly used to provide information regarding the strength of the interactions between a surface and adsorbate species, and it is often called Temperature Programmed Desorption (TPD) [9]. As temperature rises, more thermal energy becomes available. When this energy is enough to break the bond with the adsorbate, desorption is observed. At a certain temperature, the amount of the remaining adsorbed contaminant is so small that its concentration in the gas phase starts to decrease, showing a peak in the desorption spectrum. Therefore, desorption experiments are powerful tools to describe the nature of bonds between different adsorbates and substrates. 

The contamination of TCA in cork is commonly assessed by SPME-GC after a 24 h soak in a wine simulant [10]. If cork stoppers have more than 2.1 ng/L of ‘cork taint’, it can be noticed in wine by most common consumers [7]. Although the correlation between the concentration in the liquid phase (as in wine) and the concentration in the gas phase (as when we smell a cork) is unknown, the gas phase concentration is expected to be of the same order in the low pptv (part per 10^12^ by volume). This extremely low concentration of TCA released to the air by contaminated corks is well below the limit of detection of most mass spectrometers available for VOC (volatile organic compounds) analysis. Recently, some manufacturers succeeded in combining chemical ionization with time-of-flight mass analyzers to detect TCA in naturally contaminated cork samples [11,12]. For such purpose, samples were heated inside an incubation chamber at atmospheric pressure for many seconds, and then air, enriched with TCA and many other VOCs released by the cork, was introduced in the analyzer for screening.

To address the experimental challenge of studying TCA release from cork during heating, an effective strategy would be to generate samples with a higher contamination level. This would allow us to reach the contamination limit of the selected analyzer. Importantly, the interaction between TCA and cork is not anticipated to be influenced by the concentration of TCA. Assuming that the adsorption chemistry between TCA and cork remains consistent regardless of the TCA concentration, samples with high contamination levels should behave similarly, provided that the amount of TCA is kept significantly below a single TCA monolayer. This is consistent with the Langmuir adsorption model, which assumes that adsorbates primarily interact with the substrate at a submonolayer level [13].

The present work takes advantage of this approach to prepare samples. With this work, we intend to answer the following questions:

- Will TCA present a well-defined desorption peak before the cork starts to degrade?
- Can most TCA be released if the cork is heated at a well-defined temperature?
- Is TCA condensed in any form of crystals or strongly adsorbed on cork cells?

The insight gained by answering these questions is expected to support the design of curative processes to remove TCA from cork at an industrial scale. In this study, we use the term ‘adsorbed’ to describe the state of where its molecules predominantly interact with the cork substrate, regardless of whether they are located on the surface or within the cork cell walls. The term ‘condensed’ is specifically used to describe continuous forms of TCA, such as in crystalline structures.

## 2. Material and Methods

### 2.1. Sample Preparation

The samples were cut with a rotating sharp disc from natural cork stoppers in 3 mm thick slices. Cork stoppers, with 24 mm of diameter and free of TCA (nondetectable TCA), were provided by Amorim Cork, S.A. (Santa Maria de Lamas, Portugal). To make sure that the stoppers were free of TCA, releasable TCA was determined according to ISO 20752:2014 [10] (explained in Section 2.4). If the releasable TCA was below the quantification limit (0.5 ng/L), then the stopper was assumed to be free of TCA. Typically, two of these slices were used in each experiment. TCA was added by dispensing 100 µL of a 1 g/L solution of TCA (purity ≥ 99%, Aldrich, St. Louis, MO, USA) in ethanol (purity of 99.8%, Chem-Lab) on the two slices by using a micropipette. The amount of TCA was 100 µg to ensure a mass spectrometric signal with a good signal-to-noise ratio. This amount of TCA is enough to cover 283 cm^2^ with a single monolayer, considering the typical layer density of 10^15^ particles per square centimeter (which is based on simple geometric considerations). Since cork is made of hollowed cells, the real area is very large. Taking the range of dimensions of the cork cells from reference [14], the two cork discs are expected to be between 17,204 and 155,660 cm^2^. Since the ethanolic solution thoroughly wets the whole sample, upon drying, most TCA molecules were expected to be sparsely adsorbed on the cell walls, having no interaction among them (Langmuir adsorption). After contamination, the samples were left to dry for 5 min.

The advantage of using an ethanol solution of TCA over aerial exposure to a contaminated environment is that it allows for the precise control of the amount of TCA added to the cork samples. Additionally, it has been shown that most of the aerial contamination is partially removed by a soak [5]. Since one of the goals of the present work is to determine the amount of TCA extracted by heating, it is crucial to know the initial amount of added TCA. Ethanol is known to be a good solvent for TCA and quite inert to cork, so no interaction with TCA or with cork is likely.

### 2.2. Thermal Desorption Apparatus

**Figure 1:**
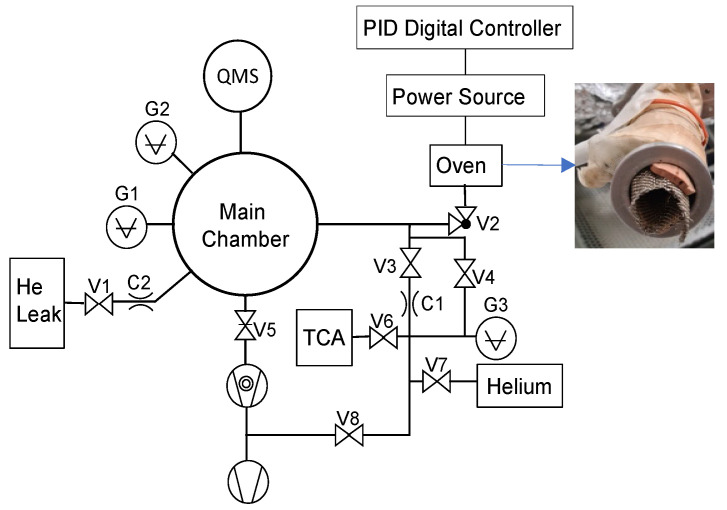
Schematic of the experimental setup for temperature-programmed desorption experiments; the photo shows one 3 mm cork disc being pressed with a net against the walls of the oven. Valves are labeled V1–V8, flow restrictions C1–C2 and vacuum gauges G1–G3.

The used setup is shown schematically in Figure 1. Desorption experiments were performed in a high vacuum (HV) chamber with a base pressure < $1\times 10^{-7}$ mbar. A spherical main chamber with a radius of 15 cm was connected via a gate valve to a Pfeiffer TPU 320 turbomolecular pump (320 L/s) backed by a dual-stage Edwards E2M2 rotary vane pump (0.71 L/s). The pressure was monitored by two vacuum gauges—a Penning ionization gauge (Balzers IKR 020 compact cold cathode gauge, G1) and a convectron gauge (Granville-Phillips, G2). 

A Balzers QMA 125-200 quadrupole mass spectrometer with an electron multiplier (SEM voltage = 2500 V) was used to monitor the evolved gas during sample heating. The quadrupole mass filter was retuned to maximize the transmission at high masses at the expense of some resolution loss (∆m ≈ 2 Th). Ions were produced by electron bombardment at 100 eV of energy. The manufacturer application Quadstar 32-bit, version 7.02, was used for control and data acquisition.

The samples were introduced in a cylindrical aluminum homemade oven wound by a heating resistor and controlled by a PID (Proportional–Integrative–Derivative) controller (Omron E5CC digital controller) connected to a power supply (ISO-TECH IPS-606D). Sample discs were pressed against the oven wall by a stainless-steel grid to improve the thermal contact. The oven was connected to the chamber by a KF25 right-angle valve and a KF25 tee vacuum fitting. The second connection of this tee was connected to a calibrated TCA gas flow used to calibrate the amount of TCA released by the cork samples. This is described in detail in the next subsection.

The vacuum chamber, mass spectrometer and tubing were wrapped with heating tapes and aluminum foil to continuously keep them at 80 °C. The heating power was adjusted by a Variac autotransformer. In this way, the adsorption of TCA on the walls was minimized as well as the adsorption of other condensable vapors released by the cork during heating.

Desorption experiments were typically performed 2 h after the introduction of samples in the oven, which was the time required to reach a working pressure below $1\times 10^{-5}$ mbar. All the samples were heated at a rate of dT/dt = 2 K/min from room temperature to ≈205 °C. The ion peak of 195 Da was followed by the mass spectrometer, which is the main peak of TCA produced by electron bombardment. The ions 197 and 199 Da were also followed and showed the same behavior. Due to the similarity of these experiments with the TPD technique described in the introduction, hereafter, the TPD acronym is used to refer to these experiments.

### 2.3. Calibration of the TCA Flow

The intensity of desorbed TCA during heating was compared with a known TCA flow generated by a lab-made reference system. Developing this system took advantage of the authors’ experience in the calibration and construction of reference leaks [15]. The lab at CEFITEC is accredited for vacuum and flow calibrations in accordance with ISO/IEC17025. The developed calibration method provided an absolute estimation of the flow rate of the desorbed TCA, which is enough for this work. Although the process described below was very rigorous, some approximations were taken as described ahead.

The calibration working principle was based on a flow restriction to introduce some TCA vapor from the headspace of a vial containing some crystals into the main chamber via the same port as the desorbed flow from the sample. A flow restriction (C1) was made by crimping a capillary metallic tube. In the first approximation, the conductance was measured for helium by using a mass spectrometer helium leak detector. Then, it was fitted to the thermal desorption apparatus as shown in Figure 1. It was connected to a small vial with some grains of TCA (<0.1 g), to the gauge G3, to a helium line and to an intermediate vacuum line. G3 was a high-accuracy capacitance diaphragm gauge (MKS Baratron 690A 1000 Torr Manometer) which provides a measurement independent of the gas type. A calibrated helium leak (VIC OM5-300) to the main chamber with a flow rate of $1.97\times 10^{-5}$ mbar. L/s was also fitted. 

To measure the conductance of C1, a known pressure of helium ($p_{He}$) (measured by G3) was generated upstream. The flow rate of helium ($Q_{He}$) was calculated by using the mass spectrometer by comparing the intensity of the ion of mass 4 Da (helium) when helium was flowing via C1 with the intensity of the reference leak. At the same time, the pressure at the main chamber ($p_{Chamber}$) was also measured by G2. 

The conductance C1 for He is then given by

(1) $$C_{He}=\frac{Q_{He}}{p_{He}-p_{Chamber}}$$

Introducing the reference flow rate from the calibrated leak ($Q_{He,\;ref}$) and the signals measured by the mass spectrometer for He via C1 (${Signal}_{He}$) and from the calibrated leak (${Signal}_{He,ref}$), the conductance for helium is given by

(2) $$C_{He}=Q_{He,\;ref}\frac{{Signal}_{He}}{{Signal}_{He,ref}\left(p_{He}-p_{Chamber}\right)}$$

Since $p_{Chamber}$ << $p_{He}$, any error in its measurement is not relevant.

If the flow regime is molecular, then the conductance is constant for the low-pressure range, and a well-known correction can be introduced to convert the helium flow rate to the TCA flow rate. To confirm the molecular flow regime, several measurements were taken by varying the helium pressure around the TCA vapor pressure. The good linear fit between $Q_{He}$ and $p_{He}$ showed that the regime was indeed molecular. Under this regime, collisions between molecules can be neglected. Therefore, the flow is only governed by the geometry and by the particles’ thermal velocity, which can be calculated from the Maxwell–Boltzmann distribution. Finally, the correction to convert $C_{He}$ to $C_{TCA}$ is

(3) $$C_{TCA}=C_{He}\sqrt{\frac{M_{He}}{M_{TCA}}}$$

Once the conductance for TCA is estimated, a known TCA flow can be generated if the TCA pressure is known. The vial with TCA was cooled with liquid nitrogen and then pumped by using the bypass to the main chamber down to a pressure of ≈$10^{-6}$ mbar. After leaving the vial at room temperature, its total pressure reached about the vapor pressure of TCA (≈$2\times 10^{-2}$ mbar). Even if the vial total pressure rises above that value, the TCA partial pressure is always the same as long as its temperature remains approximately constant.

The above procedure assumed the following approximations: the pumping speed of the turbomolecular pump is the same for He and for TCA, TCA follows the Maxwell–Boltzmann distribution, C1 is reasonably constant with temperature and with time, the TCA adsorption on the tube walls is in equilibrium with the gas phase, the mass spectrometer is linear over several decades and its time drift is small during the desorption experiments (≈2 h). Considering these approximations and the calibration uncertainties of the gauges and of the reference leak, we propose an expanded uncertainty (k = 2) for this ‘calibration’ between 30 and 40%.

Performing the above measurements and calculations three times, the reference flow rate obtained for TCA was 0.79 µg/min with a standard deviation of 3.6%. The generated signal in the mass spectrometer was well above the noise with an intensity suitable to estimate the absolute desorption rate (in µg/min) during the experiments. After the completion of each desorption experiment, the oven valve (V2) was closed, V3 was opened after the TCA signal decreased to the noise level and the TCA reference signal was recorded. To minimize the condensation of TCA inside the tubes, all the tubes were permanently kept at ≈80 °C. However, the vial with TCA was always kept at a room temperature of about 23 °C.

### 2.4. Experiments with Naturally Contaminated Samples

To evaluate the efficiency of thermal desorption on naturally contaminated stoppers, the releasable TCA was determined in the stoppers before they were subjected to a thermal process in vacuum.

Complying with the ISO 20752:2014 standard [10], each stopper was individually soaked in a 12% (*v*/*v*) hydroalcoholic solution for 24 ± 2 h. Then, a 10 mL sample was collected for analysis. The test portion was saturated with sodium chloride (NaCl, purity ≥ 99.5%), and 2,3,6-trichloroanisole (Neochema, purity ≥ 99.0%) was used as the internal standard. Ethanol had a minimum purity of 96%, and pure deionized water without TCA was used. Releasable TCA was measured by SPME-GC (solid-phase microextraction followed by gas chromatography) with an electron-capture detector (ECD) Bruker 450-GC equipped with a Combipal autosampler. Further details used for the quantification of the releasable TCA can be found in [5].

After selecting 215 cork stoppers with a releasable TCA concentration exceeding 0.5 ng/L, they were subjected to an 8 h processing period at temperatures exceeding 150 °C within a rotating drum connected to a double-stage rotary vane pump. This time was needed to reach the cork beyond its surface. The minimal pressure achieved was approximately 0.1 mbar. Then, the releasable TCA in each stopper was determined as explained before.

### 2.5. Statistical Analysis 

A basic statistical analysis was conducted in an Excel worksheet by calculating the averages and standard deviations. No further statistical analysis was required. The natural variability of the cork was the major source of measurement dispersion, as noted in several other works on cork [16,17,18].

## 3. Results and Discussion

### 3.1. Thermal Desorption of TCA

Two cork discs with a total of 100 µg of TCA were inserted in the oven and pumped down until the pressure in the main chamber was below $1\times 10^{-5}$ mbar. The samples were then heated at a rate of 2 K/min, and the peak at 195 Da was followed by the mass spectrometer as described before. 

**Figure 2:**
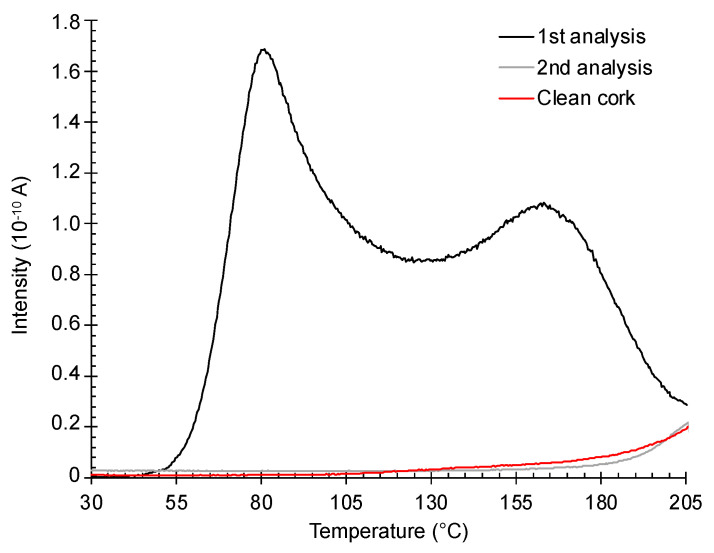
TCA desorption spectra from artificially contaminated cork and comparison with a clean cork (red curve).

The desorption spectrum is presented in Figure 2. At room temperature, no TCA was detected by the mass spectrometer, although the initial pressure was well below the TCA vapor pressure. Only at a temperature above 50 °C was some TCA detected. The spectrum shows two clear peaks, the first at ≈80 °C and the second at ≈170 °C. Above 170 °C, the desorption rate decreases continuously due to the full depletion of TCA in the cork samples. After performing a second TPD on the same samples, no further TCA was detected, meaning that most TCA was released (grey curve of Figure 2).

When TPD was performed on the samples without TCA (red curve in Figure 2), a similar trend could be perceived. However, while the black and grey curves are from the same sample, the red curve is from another cork sample.

The small rise after 180 °C was due to the degradation of cork and not the presence of extra TCA, which could be released at higher temperatures. Experiments conducted at temperatures up to 250 °C revealed a decline in the signal of the 195 Da ion, with no observable peak. At the same time, many more ion peaks could be seen in the mass spectrum. These data are omitted to keep the focus of the present work.

A thermogravimetric analysis of the cork showed that the cork withstands a temperature of up to ≈180 °C without major damage. The loss of about 6% in mass was due to the release of water [14]. Şen and coauthors confirmed that above 180 °C, cork starts to degrade [19]; this is when acetic acid and furfural begin to be released due to the degradation of polysaccharides, which are the first structural components to be thermally degraded [20,21]. Beyond this temperature, the mass loss increases until ashing at 485 °C.

### 3.2. Quantification of Desorbed TCA

**Figure 3:**
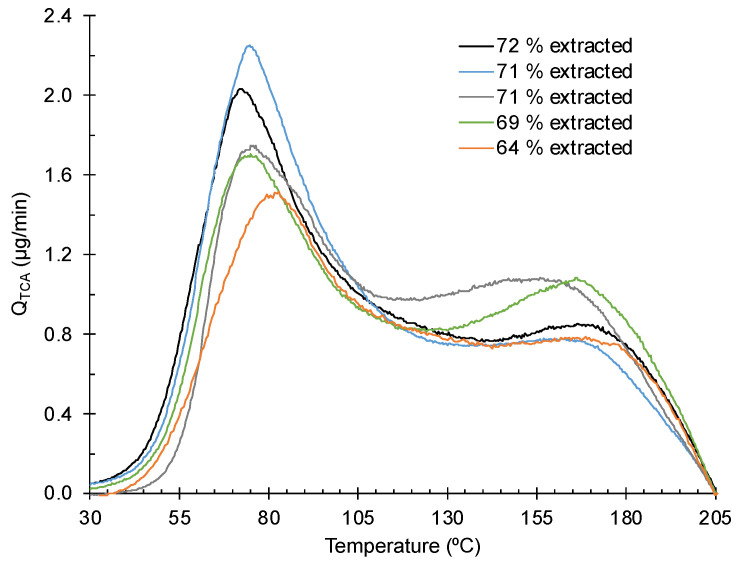
TCA desorption spectra after 2 h under vacuum of five samples. The intensity scale was converted to mass flow rate. The legend shows the relative amount of extracted TCA.

Once the TCA reference flow is known, the signal of the desorbed TCA can be converted to an absolute mass flow rate as shown in Figure 3 for five different samples. In these plots, the background signal was removed by subtracting the signal from a clean cork sample after adjusting its intensity at 205 °C to have the same value for each sample with TCA. Thus, all the plots are forced to display an intensity of zero at 205 °C.

Since the time is proportional to the temperature, the area under each curve returns the total amount of released TCA after dividing the corresponding integral with the heating rate. The latter can be compared with the amount of the TCA added to each sample. Figure 3 shows that the released TCA is always lower than that added. For the five samples, the average is 69 ± 3%. The difference of ≈31% is attributed to the sublimation of the condensed TCA on the cork surface formed when the solvent evaporates, which occurs before starting to heat the samples.

The evaporation (or sublimation) rate of a substance can be estimated by its vapor pressure and temperature from a balance between the evaporation and condensation [22]. When the concentration in the vapor phase is much lower than that in the saturation conditions, condensation can be neglected. Under these conditions, taking the TCA vapor pressure of 3.1 Pa at 20 °C [23], the evaporation rate is 3.35 µg/cm^2^/min. Since we added 100 µg of TCA to each sample, most condensed TCA in the form of crystals on the surface would be evaporated on a scale of minutes. This is confirmed by a simple experiment in which grains of TCA left exposed to air sublimate quickly in that time scale.

All the desorption curves showed the presence of the same two peaks (cf. Figure 2). Some variability in the peak position can be seen as well as in its intensities. This kind of variation is common in cork experiments and is due to the natural variability of the cork density, chemical composition and cell dimensions.

### 3.3. Effect of Elapsed Time after Sample Preparation

**Figure 4:**
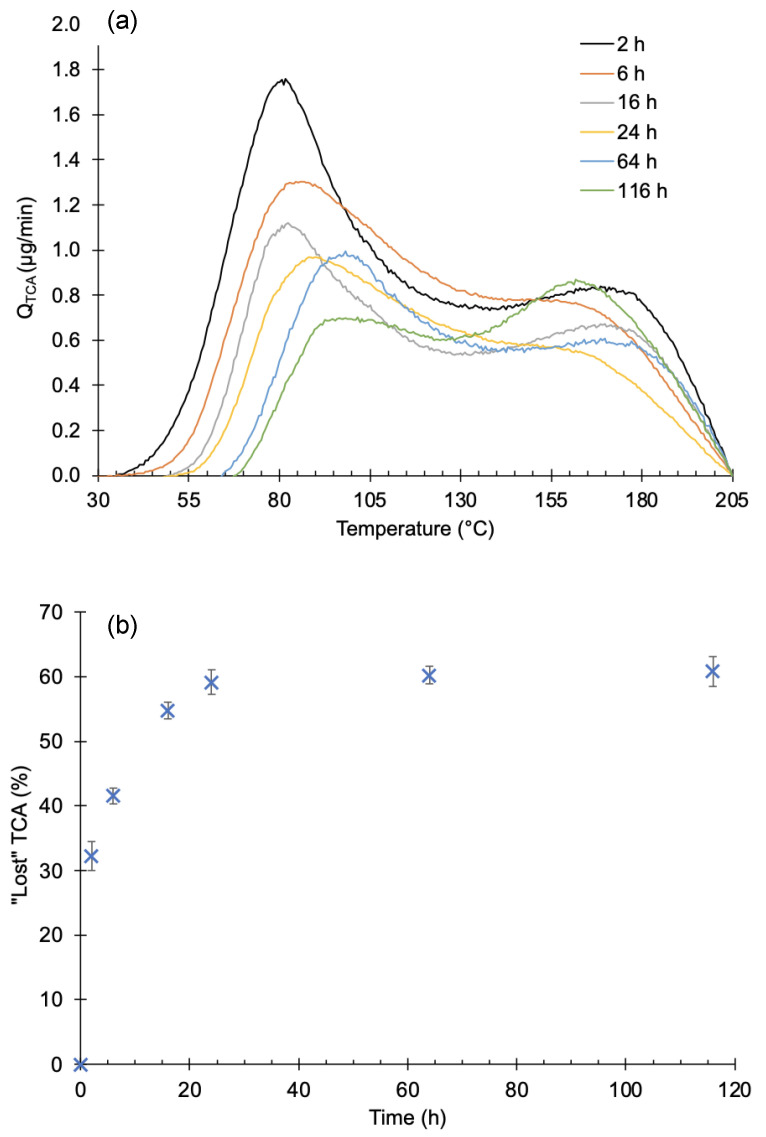
The influence of vacuum on contaminated samples studied over time. (**a**) TCA desorption spectra on contaminated samples that stayed under high vacuum. Each curve is the average of 3 measurements. (**b**) Plot of the “lost” TCA in contaminated samples over time. Each point in (**b**) is the average of the area from 3 experiments, and the error bars are the standard deviation.

To evaluate the effect of time and pressure on the release of TCA, two sets of experiments were performed. In the first set, the time that samples were under high vacuum prior to heating was varied. Results are shown in Figure 4a for times between 2 and 116 h (≈5 days). Each curve represents the average of three measurements. The area of the first peak decreased with time and its maximum was shifted towards higher temperatures. There is not a clear trend for the second peak. The observed variation is attributed to the natural variation in the material, as in Figure 3. The absence of the left part of the curve, between 40 and 75 °C, indicates that a part of TCA was released before heating. After 20 h under vacuum, approximately 60% of the added TCA was removed, as shown in Figure 4b. After this time, the removed TCA slowly changes.

**Figure 5:**
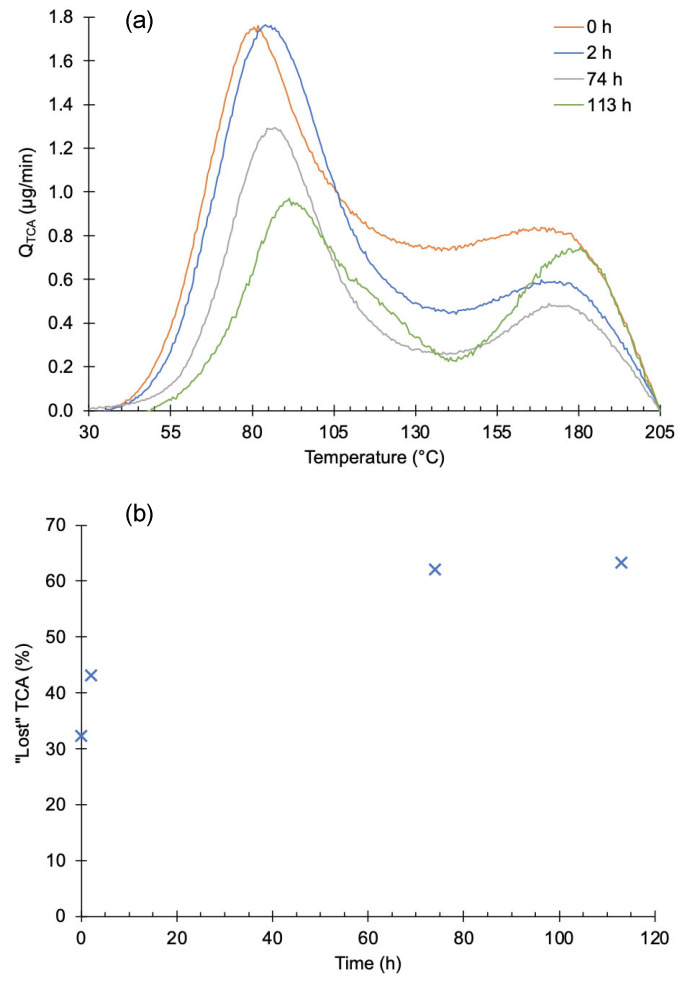
Study of the influence of time between contamination and analysis. (**a**) TCA desorption spectra of contaminated samples that stayed at atmospheric pressure in a vented space. (**b**) Plot of “lost” TCA; all the samples stayed for 2 h in vacuum to reach the working pressure before TPD.

Another set of experiments was conducted by leaving the samples at atmospheric pressure in a vented space. Results are depicted in Figure 5. Although only one measurement was performed each time, the same trend is observed, with a significant loss of TCA from the first peak. The second peak is preserved under a vacuum. The amount of ‘lost TCA’ was about the same as that in the previous experiments.

Neither of the two temperature peaks should be assigned to a phase change (sublimation) since all the samples were well below the TCA vapor pressure for some hours before heating. This time was more than enough to vaporize any condensed TCA on the sample surface. Therefore, due to the different effects of time on these peaks, we assign the first to gas-phase transport throughout the cork and the second to desorption from the cork cell walls.

It is relevant to remind readers that cork consists of hollowed cells in a honeycomb-like arrangement with air inside. These cells are quite tight, which is the reason for their application in stoppers. Nevertheless, cork cells are slowly pumped down to very low pressures at a predictable rate, as shown in previous work [24]. Once TCA is added to cork, its vapor can permeate the cell walls and move across the sample. Upon sample preparation, the ethanolic TCA solution is poured over the sample, wetting the whole piece of cork. Some TCA penetrates through cell walls, taking the TCA inside. Therefore, we propose the formation of three different states of TCA in cork after solvent evaporation:

- Condensed TCA in the form of tiny crystals, mainly on the cork surface;
- TCA vapor enclosed inside the cork cells;
- TCA bonded to the cork cells, either on their surface or throughout the whole sample.

The condensed TCA could be formed in spots where more solvent was evaporated, closer to the surface. This TCA quickly sublimates and leaves the sample, as discussed before. If crystals are formed inside closed cells, then they will reach an equilibrium with the vapor phase inside those cells. Such TCA vapor will diffuse towards the cells with a lower concentration or to the external surface and then be removed by the vacuum pumps or dispersed in the air. This process is strongly time-dependent since it is governed by Fick’s laws of diffusion. The change in the shape of the first peak over time is produced by the gradual loss of the TCA vapor in cells near the surface at room temperature. The longer the time, the deeper the region from which TCA is depleted. Then, when samples are heated up, less TCA is extracted because of the previously exhausted TCA. This produces an apparent shift in temperature which only corresponds to a time delay of TCA on its way towards the surface. 

The behavior of the second peak is quite different and is assigned to TCA adsorbed on the cell walls. Its area seems to be invariant with time and exposure to a vacuum below the vapor pressure. These TCA molecules are directly bonded to the cell walls and not to other similar molecules as they are in crystals. To be released, they must overcome the adsorption energy. Then, a balance between desorption, readsorption and diffusion takes place. At a certain temperature, the desorption rate is so high that most TCA is in the gas phase. At the same time, gas diffusion quickly increases and TCA moves outside, being released from the sample.

**Figure 6:**
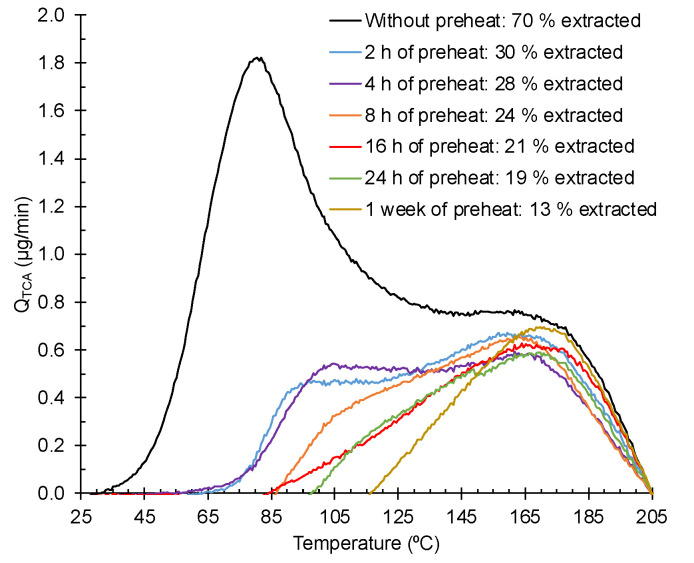
Study of preheating on contaminated samples. All samples stayed for 2 h in vacuum to reach the working pressure before the TPD.

Since diffusion is also strongly temperature-dependent, preheating samples at atmospheric pressure and moderate temperature should change the contribution assigned to diffusion. At increased temperatures, the TCA vapor diffuses faster within the sample to the outside where the concentration is lower. Less TCA in the vapor phase is expected to remain in the cork, which corresponds to the first TPD peak. On the other hand, the second peak should be unchangeable. An experiment was performed by preheating samples in an oven at 50 °C for controlled times. Figure 6 shows the results for 2, 4, 8, 16, 24 h and 1 week of preheating time. The TPD for a sample without preheating is also shown. Over time, the gradual disappearance of the first peak is clear. After several hours of preheating, only the second peak remained, becoming apparently constant for longer times. Thus, it looks consistent with the assignment of the second peak to a bonded state of TCA on the cork cell walls.

The area obtained from the 1 week curve represents about 13% of the TCA added to the sample. Therefore, although most of the TCA was released over time at a moderate temperature, still a significant amount remained strongly trapped in the cork. Only at a very high temperature could this form of TCA be removed from the cork. 

In model TPD experiments (the desorption of submonolayers from well-defined surfaces under an ultrahigh vacuum), the adsorption energy can be estimated from the position (temperature) of the peak as proposed by Redhead and often used in TPD experiments [25]. Assuming a first-order process in which molecules are desorbed without dissociation, the estimated adsorption energy is 134 kJ/mol. This energy is well above the vaporization and sublimation enthalpies, which are approximately 60 kJ/mol and 83 kJ/mol, respectively (the enthalpies were calculated by using the melting and boiling temperatures of TCA given in [23]). However, this estimation does not consider diffusion across cell walls as well as the physical and chemical complexity of the substrate. This energy leads to a time constant (corresponding to the time needed to reduce the concentration to 37% of the initial value) of many thousands of years. Hence, it supports the common knowledge that TCA contamination in cork is long-lasting and very difficult to remove in ambient conditions. 

### 3.4. Experiments with Naturally Contaminated Samples

Investigating the desorption of naturally contaminated samples poses significant technical challenges, as mentioned in the introduction. The release of TCA from such samples, even under heating, yields such a low flux that it surpasses the quantification limits of currently available instruments unless some preconcentration technique is employed. Given the time required for preconcentration, this option becomes unsuitable for performing a thermal desorption analysis. Thus, validating the aforementioned assumptions through similar experiments with naturally contaminated stoppers is not straightforward. However, additional experiments were conducted to determine whether TCA remains in stoppers after heating at a temperature close to the desorption peak.

About 44% of the 215 positive samples used for this experiment exhibited a releasable TCA concentration above 5 ng/L, with five samples between 50 and 60 ng/L. Following the processing period, no samples registered above 4.5 ng/L. The average TCA concentration decreased from 11.03 ng/L to 0.41 ng/L, indicating a 27-fold reduction. Moreover, after the process, 70% of the 215 samples registered less than 0.5 ng/L, which is the typical quantification limit for this technique. Additional experiments demonstrated that the process temperature was the critical factor in achieving a satisfactory extraction efficiency. This knowledge culminated in the filing of patents [26,27], outlining a process for extracting TCA from cork stoppers on an industrial scale, which is currently being utilized in the production of natural cork stoppers (stoppers made from a single piece of cork). These patents provide further details on the effect of time, pressure and temperature on the extraction efficiency.

## 4. Conclusions

The thermal desorption of TCA was successfully performed in artificially contaminated samples. These samples had enough TCA to be detected by a quadrupole mass analyzer with an electron impact ion source. When the samples were preheated at 50 °C for many hours, the spectra showed just one desorption peak. Even if the contamination process did not fully replicate the natural process, it seems reasonable to believe that naturally contaminated cork should have a similar spectrum (with a single peak) since planks are boiled in water and harvested long before the stoppers are manufactured.

A calibration technique was developed to provide the absolute desorption rate. The calibrated spectra showed that the amount of released TCA was about the same as that added to the samples. Moreover, no TCA was detected when the samples were heated for a second time. Thus, heating at high temperatures was suitable to extract most TCA from the cork, even if they were strongly bonded. This was confirmed with naturally contaminated samples in which a high degree of extraction was achieved.

The proposed technique appears to be an important tool for understanding how TCA is bonded in cork. An energy of 134 kJ/mol is proposed as a first estimation of the adsorption energy of TCA on the cork. Nonetheless, considering the chemical complexity of cork, further investigation is necessary to understand which of its primary components is more susceptible to TCA contamination.

## 5. Patent

Patent # WO 2018/138599 A1, Patent #PT109878B.

## Data Availability

All the data supporting the findings of this study are available in the article.
